# The Single Incision Minimally Invasive (SIMI) Neck Lift

**DOI:** 10.1097/GOX.0000000000002208

**Published:** 2019-05-16

**Authors:** Jonathan L. Kaplan

**Affiliations:** From the * Department of Plastic Surgery, Dermatology and Plastic Surgery Institute, Cleveland Clinic, Cleveland, Ohio; † Pacific Heights Plastic Surgery, San Francisco, Calif.

## Abstract

**Background:**

With the increased focus on the neck and chin in the era of selfies and social media, neck contouring continues to be an in-demand procedure. Full correction of the neck typically requires both submental and postauricular incisions, but the postauricular incisions can be unsightly and painful. This article introduces a viable alternative for the patient.

**Methods:**

A case series of appropriate candidates undergoing a novel neck lift technique (the single incision minimally invasive neck lift) is described. Participants include men and women with an age range of 30–70. The surgical technique incorporates shifting of the typical submental incision used in a full neck lift to a cervicomental incision, and when accompanied by wide undermining, the postauricular incisions are avoided entirely.

**Results:**

Among the 20 patients in this case study, photographs show that it is possible to achieve results commonly associated with a full neck lift but without the postauricular incisions typically associated with a full neck lift.

**Conclusions:**

The single incision minimally invasive neck lift allows the surgeon to offer an effective procedure for appropriate candidates who want to treat excess fat, a sagging platysma muscle and loose, misplaced skin in the submental region without postauricular incisions typically utilized in a traditional full neck lift.

## Abstract 2

Supplemental Digital Content is available in the text.

## INTRODUCTION

Neck contouring continues to be a very sought-after procedure in the era of social media and selfies.^1^ Awareness of these procedures via marketing and social media generates interest within the Millennials (born 1981–1996, age 22–37 years in 2018) and Generation X (born 1965–1980, age 38–53 years in 2018) communities, whereas traditionally, patients who consider neck lifts fit within the Baby Boomers (born 1946–1964, age 54–72 years in 2018) age bracket.^2^

With this changing demographic come different expectations. In addition to seeking out completely noninvasive procedures, they are considering minimally invasive procedures. Deciding factors include length of work and/or social down time, placement and concealment of incisions, number of incisions, and the risk–reward benefit of undergoing the procedure.

To meet the demand of these changing expectations, surgeons must innovate to attract this clientele. In the past, a full neck lift was necessary to achieve long-lasting results. However, younger patients may be more reluctant to have an invasive procedure that requires both submental and postauricular incisions.

During a cosmetic surgery consultation, the Pythagorean theorem and Euclidean geometry^3^ are used as a guide to determine appropriate candidates who will achieve the same results as a traditional neck lift with a novel type of "mini" neck lift. This case series discusses the single incision minimally invasive (SIMI) neck lift and how consistent reproducible results are achievable through 1 incision instead of 3.

## PATIENTS AND METHODS

This case series includes 20 consecutive patients who underwent a SIMI neck lift. The patients provided informed consent, and several patients allowed the primary surgeon and author (JLK) to record the procedure on Snapchat and Instagram as is standard in the author’s practice. Patients also agreed for their photographs to be used in this case study and for marketing purposes.

Patients with “misplaced” midline neck skin, without a great deal of excess skin, were considered candidates for this modified neck lift. The 20 patients included in this study underwent surgery between May 2015 and August 2018 (approximately 3.5 years). They were fairly evenly divided between males and females (11 women and 9 men) with an average age of 55 (range 30–70) years (Table 1).

**Table 1.**
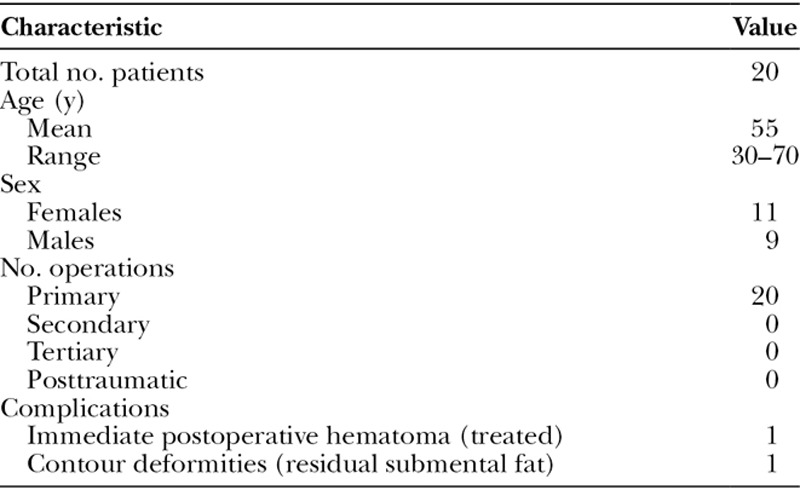
Patient Demographics and Complications

### Operative Technique

In the preoperative holding area, the patient is marked. A 3–4 cm line is drawn on the radial side of the left index finger and then superimposed on the junction of the horizontal and vertical surface of the neck (the cervicomental angle) in a transverse direction. Using this technique allows the surgeon to feel for that junction, thus ensuring that the incision is placed in the most hidden, concave surface of the cervicomental angle of the neck, within the neck’s natural shadow (**see** video, Supplemental Digital Content 1, which shows appropriate preoperative marking of the ideal SIMI neck lift candidate, intraoperative technique, and reproducible postoperative results. This video is available in the “Related Videos” section of the Full-Text article on PRSjournal.com or at http://links.lww.com/PRSGO/B71).

**Video Graphic 1.**
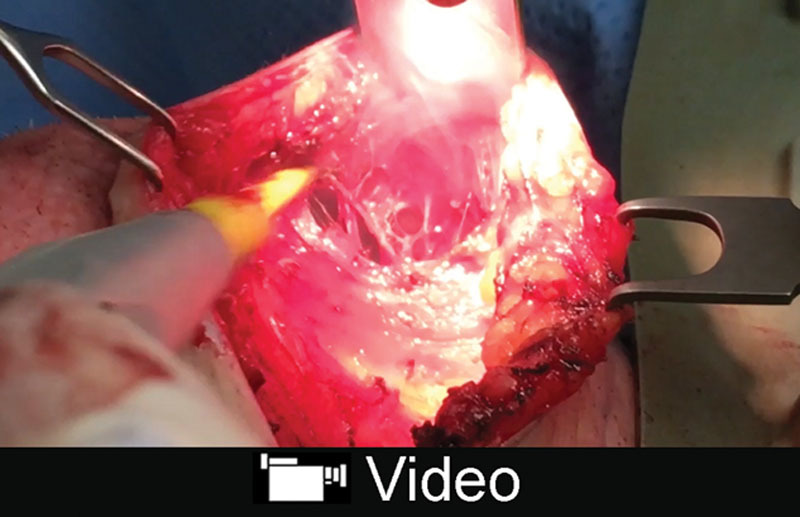
See video, Supplemental Digital Content 1, which shows appropriate preoperative marking of the ideal SIMI neck lift candidate, intraoperative technique, and reproducible postoperative results. This video is available in the “Related Videos” section of the Full-Text article on PRSGlobalOpen.com or at http://links.lww.com/PRSGO/B71.

The lateral extent of the neck dissection is marked along the edge of the sternocleidomastoid, with the superior most dissection marked along the jawline and the inferior most dissection marked along the base of the neck. The 3–4 cm incision allows full access for complete dissection of these areas.

In the operating room, the patient is given a standard dose of 1 g of cefazolin and SCDs are placed. This is a procedure that can be done under both conscious sedation and general anesthesia.

A stab incision is made in the center of the transverse preoperative marking in the cervicomental angle of the neck with a # 15 blade. A total of 250 ml of tumescent solution is infiltrated under low flow. Tumescent solution consists of saline and 1 amp of epinephrine (no lidocaine) if under general or 50 ml of 1% lidocaine and 1 amp of epinephrine if performed under conscious sedation. After infiltration, 7–10 minutes is allowed to elapse for maximum vasoconstriction.

Liposuction with a 3-mm cannula is performed along the entirety of the neck, extending to the sternocleidomastoid and especially along the jawline to reduce any jowling that is present. After liposuction, the remainder of the transverse neck incision is completed.

Under direct visualization with a lighted retractor, the neck skin is elevated bilaterally to the sternocleidomastoid, anteriorly toward the chin, and inferiorly toward the base of the neck. Because there are no postauricular incisions in the SIMI neck lift, the surgeon must widely undermine and reposition the misplaced skin.

After elevation and hemostasis, the medial edges of the platysma are delineated in preparation for the corset platysmaplasty as described by Feldman.^4^ Any subplatysmal fat is excised down to the level of the digastric muscles. A 4–0 PDS is run from the superior aspect of the platysma to the base of the neck and back up in a running horizontal mattress fashion to minimize the appearance or palpation of a midline ridge.

Any excess fat present just posterior to the chin or attached to the undersurface of the skin flap is directly excised. One 10 French round drain is brought through a separate stab incision behind either earlobe and placed across the entire neck. Only one drain is used and is sewn into place with leftover 4–0 PDS used in the corset platysmaplasty.

The cervicomental incision is closed with 6–0 fast absorbing gut along the skin edges in a baseball stitch fashion (simple continuous or running suture technique). No dermal stitch is required. The patient is placed into a postoperative head and neck garment and transferred to the recovery room. Patients are sent home with an extra garment and an already filled prescription of pain medication, antiemetics, and silicone scar cream. The silicone scar cream can be applied 2 weeks after procedure and is recommended to further minimize the appearance of the scar by increasing collagen production.^5^

The patient is seen the next morning (postoperative day one) to remove the drain. Showers commence the next morning after drain removal. The garment is worn all of the time during the first week except during showers and then only at night for several more weeks.

Because the only incision is closed with absorbable stitches, the patient does not have to return for suture removal, other than the drain stitch on postoperative day 1. The patient returns for a checkup and postoperative photographs at 2 weeks and 3–4 months.

## RESULTS

Twenty patients underwent a SIMI neck lift. These were primary cases. Patients who were considered candidates for the SIMI neck lift were those with excess fat and skin to the neck and reflect the SIMI neck lift pathway in the decision tree found in Figure 8.

The only observed complication was an immediate postoperative hematoma that was noted in the recovery room. The patient was brought back to the operating room, and the operative site was explored through the existing cervicomental incision. No vessel was identified other than generalized ooze. Hemostasis was achieved without the need for additional incisions, and the patient had an uncomplicated recovery from that point onward.

One patient had residual submental fat remaining after his SIMI neck lift. Dissolution of the fat with deoxycholic acid was offered, but the patient was happy with his result and declined.

Each figure shows results at 3–4 months postoperative. The impetus for doing a SIMI neck lift, or why a SIMI neck lift was more appropriate than another technique, is clarified with each before and after result.

### Case # 1

The patient in case # 1 was a perfect candidate for a SIMI neck lift, because he had minimal excess skin and fat. However, a lax platysma accentuated the excess skin and fat, giving him a “double chin” appearance.

Due to this lax platysma, liposuction alone would not achieve his desired result. A traditional mini neck lift through a submental incision would not allow for the wide undermining necessary to redrape the skin and once the underlying platysma muscle is tightened, the skin would continue to hang or buckle along the edge of the nonundermined areas. A full neck lift requiring postauricular incisions would achieve the same result as achieved by the SIMI neck lift. But with no excess skin to treat or remove, the additional postauricular incisions would have been unnecessary and too visible with the patient’s short hair.

**Fig. 1.**
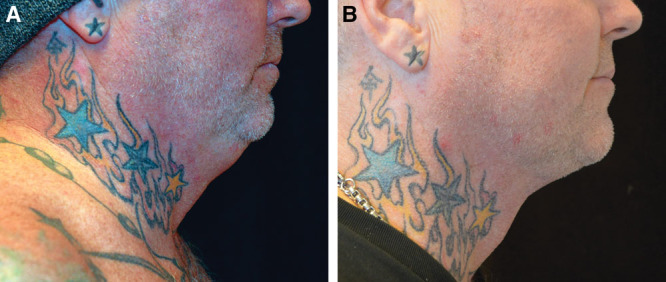
Patient # 1 before and after results at 3 months.

As seen in Figure 1, the patient’s results improved during the first 3 months postoperatively as the redraped skin continued to tighten. His chin appears more prominent and he no longer has the double chin appearance.

### Case # 2

The SIMI neck lift was appropriate for patient # 2, because she had misplaced midline skin.

She did not have excess fat, so liposuction alone would not have adequately treated her. Some skin redraping would occur with liposuction alone, but the surgeon has greater control in skin redraping by manually undermining skin rather than depending on the redraping from liposuction alone. And because this patient has misplaced midline skin, not excess skin, skin excision was not necessary and, therefore, postauricular incisions were not necessary.

**Fig. 2.**
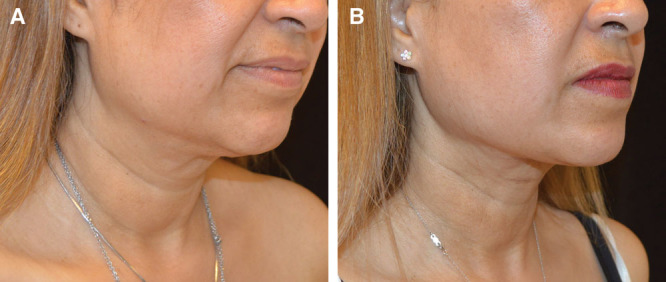
Patient # 2 before and after results at 3 months.

As seen in Figure 2, the patient’s jawline is more well defined after being treated with the SIMI neck lift. Once the skin was undermined and redraped, the skin is no longer loose and the neck appears thinner. The transverse lines present pre- and postoperatively are natural etched-in lines that are not treated with any neck lift technique.

### Case # 3

Patient # 3 was a perfect candidate for a SIMI neck lift for reasons similar to patient # 1. A very lax platysma and excess midline skin obscured the patient’s thyroid cartilage, a hallmark of masculinity. This contour was restored with his procedure.

As a male patient with short hair, postauricular incisions are difficult to hide. Performing a SIMI neck lift to redrape the skin through a cervicomental incision was most appropriate in avoiding postauricular incisions. As stated previously, the submental incision used in the traditional mini neck lift would not allow for wide undermining necessary for adequate skin redraping.

**Fig. 3.**
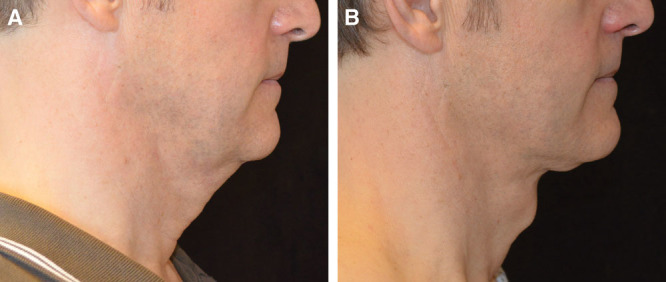
Patient # 3 before and after results at 3 months.

Similar to patient # 1, as seen in Figure 3, once the patient’s platysma was tightened and the hanging skin in the neck was undermined and redraped, the contours of his neck became more prominent. Before the surgery, the patient’s thyroid cartilage was not visible and the redraping of the skin exposed it.

This patient had residual submental fat after his procedure, but he declined to do anything further, because he was pleased with his result.

### Case # 4

Patient # 4 had a significant amount of midline excess skin. This patient could easily be a candidate for a full neck lift utilizing a submental incision and postauricular incisions. Although she could more easily cover incisions behind the ears with longer hair, she did not want the pain associated with postauricular incisions. Therefore, she opted for a SIMI neck lift.

**Fig. 4.**
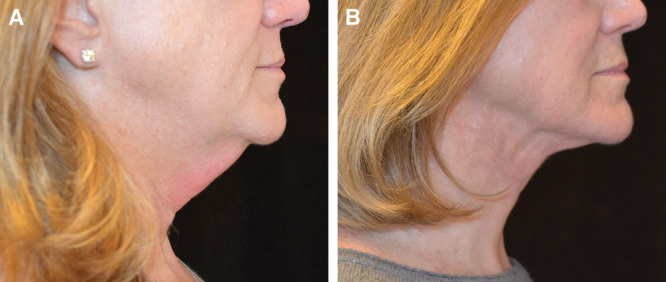
Patient # 3 before and after results at 3 months.

With so much excess skin, she was at risk of wrinkling or folding of the redraped skin, if there were no adequate recoil and tightening. At the 2-week mark, she did indeed have a fold of skin resulting in a crease, but this resolved over time and is no longer present in her 3-month postoperative photograph, as shown in Figure 4. There are subtle striations seen on the skin surface, but there are no folds, wrinkles, or creases remaining.

### Case # 5

**Fig. 5.**
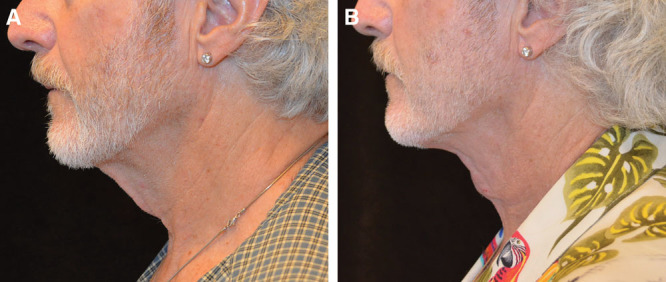
Patient # 5 before and after results at 4 months.

Patient # 5 had hanging skin that covered his laryngeal prominence. Because this skin was not in excess, he was a candidate for a SIMI neck lift. Redraping of the skin gave him more prominent chin profile. Additionally, his laryngeal prominence is more noticeable (Fig. 5).

### Case # 6

**Fig. 6.**
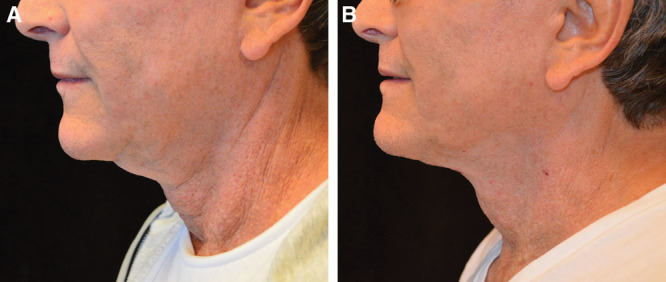
Patient # 6 before and after results at 6 weeks.

Patient # 6 was a candidate for a SIMI neck lift, because he had loose skin underneath his chin and on the lateral sides of his neck starting below his ears. Because this loose skin had minimal fat, he would not be a candidate for liposuction alone. The wide undermining of the skin during the SIMI neck lift procedure allowed all of the misplaced, bunched-up areas of skin to be redraped and smoothed out, giving his neck a more youthful appearance (Fig. 6).

## DISCUSSION

The introduction of a new technique such as the SIMI neck lift^6^ requires a review of the “benchmark” procedures that it builds upon (Table 2). This also requires an agreement on the terminology used when referring to those benchmark procedures. For example, we cannot reference a full neck lift and a mini neck lift without clarifying what those procedures entail, because those terms mean different things to different surgeons. And although we will not necessarily agree on the terminology in every context, we can agree on the terminology, at least temporarily, within the confines of this case series.

**Table 2.**
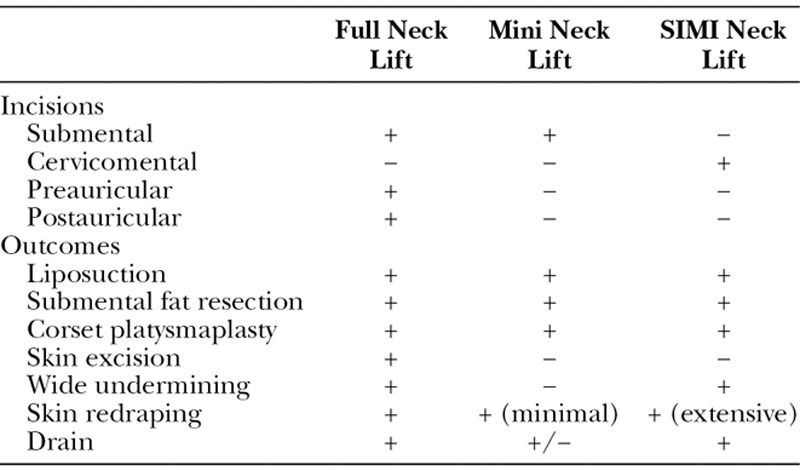
Full Neck Lift Versus Mini Neck Lift Versus SIMI Neck Lift: Comparison of Traditional Neck Lift Techniques With the Novel SIMI Neck Lift

Therefore, for the sake of this discussion, a full neck lift refers to a procedure, wherein incisions are made to the submental and postauricular areas followed by liposuction, submental fat resection, wide undermining of skin, corset platysmaplasty, and skin excision from the postauricular area.

A mini neck lift is more conservative by nature. This procedure as described by Knize^7^ requires a submental incision, just behind the chin, to remove submental fat and possibly a corset platysmaplasty.

Based on this terminology, the SIMI neck lift technique is different in regard to:

1. Incision placement: in the SIMI neck lift, the incision is more posterior, moving the traditional incision from a submental to a cervicomental location to allow greater access to the neck.
2. Much more extensive undermining of the skin. The goal of the SIMI neck lift, compared with the mini neck lift, is the redraping of the misplaced skin. Moving the submental incision to a cervicomental position in an SIMI neck lift allows for the wide undermining achieved during a full neck lift without requiring any postauricular incisions.

Using the terminology, misplaced, is meant to describe a normal amount of skin that is simply misplaced in the midline of the neck. By redraping the skin, it is repositioned to a more aesthetically pleasing position. Because there is no “excess, hanging” skin, postauricular incisions are not needed to excise this skin (Table 2).

### The Pythagorean Theorem as Part of the SIMI Neck Lift

**Fig. 7.**
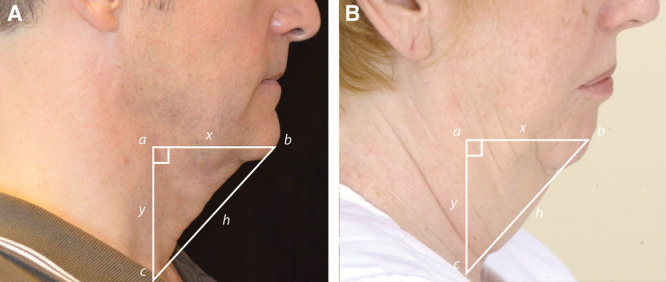
Right angle triangle superimposed on neck.

To rely solely on skin redraping requires an understanding of the Pythagorean theorem when choosing appropriate candidates for the SIMI neck lift. As shown in Figure 7, the right angle triangle is labeled *x* and *y* to represent the 2 shorter sides of the triangle (the catheti), connected by the hypotenuse, *h*, the longer line opposite the right angle. *x* represents line segment 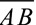, *y* represents line segment 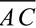, and *h* represents line segment 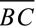. 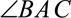 is the right angle and is equal to 90°. Based on the Phythagorean theorem, the square of the two catheti is equal to the square of the hypotenuse, 
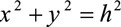
. Therefore, the sum of the two catheti is always greater than the length of the hypotenuse, 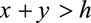.

**Fig. 8.**
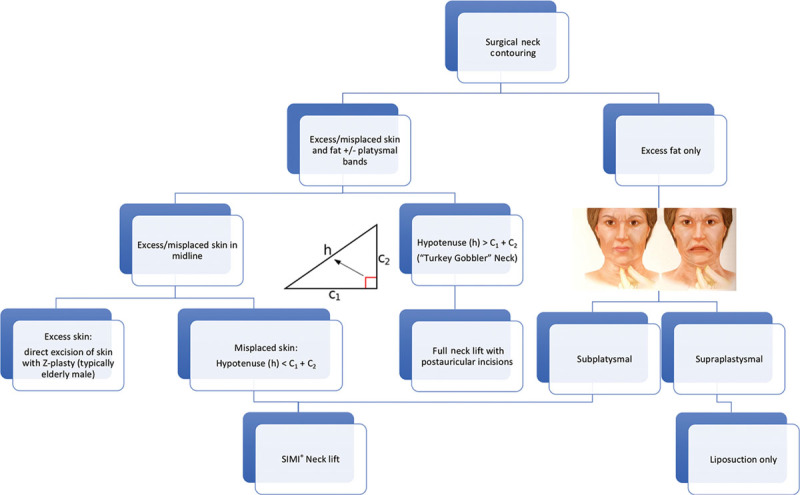
SIMI neck lift decision tree. This decision tree delineates which patients are candidates for a full neck lift, liposuction only, direct excision with Z-plasty, or the SIMI neck lift. Image showing the test for supra- vs subplatysmal fat is used courtesy of *The Art of Aesthetic Surgery*.^8^

Based on this geometry, any misplaced or “hanging” skin that is equal to or less than the hypotenuse can be accommodated within the natural contours of the neck, in line with the two catheti. When the right angle triangle is superimposed onto the neck as shown in Figure 8 below, *x* and *y* represent the mandibular and cervical portions, respectively, of the neck and the hypotenuse represents the lax, obtuse cervicomandibular angle.

Furthermore, the Vertex a is where the incision is placed at the crux of the neck or the junction of the horizontal and vertical surfaces of the neck (the cervicomental angle). Additionally, because this is the location of the transverse incision, this gives the surgeon the most access for the wide undermining described in the operative technique. Vertex b is located at the mandibular prominence to form the imaginary line segment 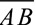. Lastly, Vertex c is placed at the suprasternal notch to form the imaginary line segment 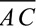. From these two points, the imaginary line segment 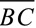 can be drawn to form our hypotenuse (Fig. 7).

As previously discussed, based on the Pythagorean theorem, the sum of the lengths of the 2 catheti is always greater than the length of the hypotenuse, 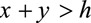. Therefore, in appropriate candidates, when the cervicomandicular angle aligns with the hypotenuse, the lax skin (the hypotenuse in this analogy) can be redraped and accommodated within the limbs (the catheti) overlying the mandibular and cervical portions of the neck. When represented by an equation, a patient is an excellent candidate for an SIMI neck lift when: 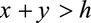. As shown in Figure 8, if the excess (or misplaced) skin does not pass the hypotenuse line of the superimposed right triangle, the patient’s natural neck contours can smoothly accommodate the skin once undermined and redraped. When candidates met these criteria, they were recommended for the SIMI neck lift.

However, if the patient has a great deal of excess skin, wherein the hypotenuse is greater than the mandibular and cervical limbs of the right-angle triangle, ie, the 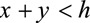, this type of patient is not a candidate for the SIMI neck lift, because the excess skin cannot be accommodated along the jawline and neck. A patient with excess skin that hangs past the line of the hypotenuse of this right triangle would require skin excision and would be a more appropriate candidate for a full neck lift.

As shown in Figure 8, patients can be separated into varying treatment pathways based on skin excess, fat excess, or platysmal banding. Patients with supraplatysmal fat only (no excess skin) are candidates for liposuction alone.^9^ Patients with subplatysmal fat, platysmal banding, or redundant skin can be further divided based on excess skin (direct excision with Z-plasty as described by Zins or a full neck lift)^10,11^ or misplaced skin (SIMI neck lift).

### Benefits of the SIMI Neck Lift

When compared with the full neck lift, the SIMI neck lift avoids painful or unsightly postauricular scars. Although less painful incisions benefit both males and females, the lack of scars behind the ears are particularly beneficial for men, because they typically have short-cropped hair, making it more difficult to hide incisions in this area. Although pain from postauricular incisions can vary between surgical technique and a patient’s pain threshold, clearly the *absence* of a postauricular incision will lead to less pain than the *presence* of a postauricular incision.

With only a single cervicomental incision, this is clearly a less invasive procedure compared with its “full neck lift” counterpart. The more posteriorly placed cervicomental incision provides easier access to the entire neck, making wide skin undermining easier. Performing the entire operation through the cervicomental incision avoids not only postauricular incisions but also the need for postauricular skin flap elevation. Wide undermining and single incision placement are the key distinguishing factors of the SIMI neck lift when compared with the previously described, “limited incision submental lipectomy and platysmaplasty” by Knize.^7^

In regard to the Knize^7^ cohort, it is important to note that our cohort of 20 patients over 3.5 years (5.7 patients per anum**) is comparable with his cohort of 56 patients over 15 years (3.7 patients per anum).

Additional benefits of the SIMI neck lift include fewer incisions, which can lead to shorter operative times due to less time required to close postauricular incisions. A reasonable assumption is that fewer incisions will lead to less pain and, therefore, a shorter recovery when compared with the full neck lift. Certainly, a prospective cohort study can further evaluate these potential advantages.

**Fig. 9.**
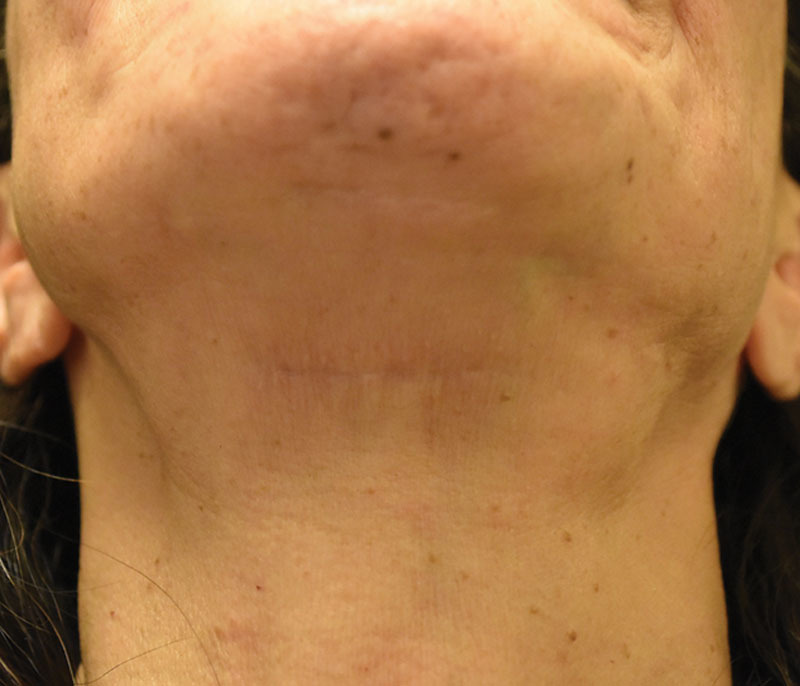
SIMI neck lift incision. The SIMI neck lift incision is hidden in the cervicomental angle of the neck. Here is an example of its appearance 15 months after a SIMI neck lift. The scar is comparable to the patient’s much older submental incision from her facelift 20 years prior, by another surgeon, but most importantly, this worm’s eye view is not the view of the neck seen in everyday social situations.

**Fig. 10.**
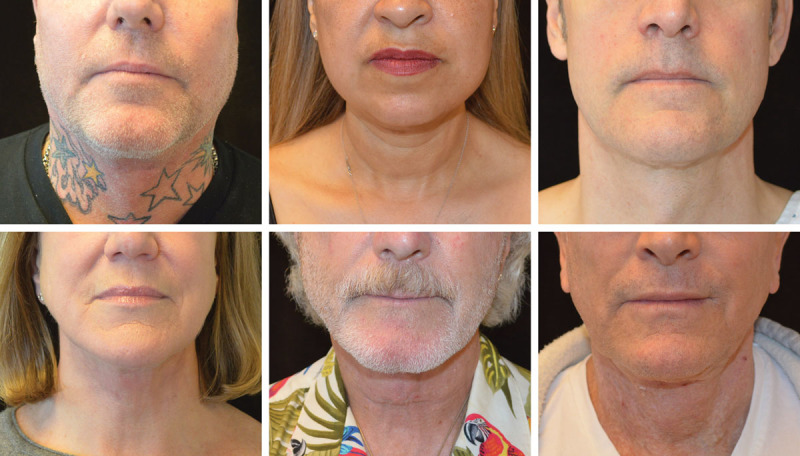
Frontal view of SIMI neck lift incision at 3–4 months postoperatively. Frontal view of all 6 patients highlighted in this case series, showing that the cervicomental incision is imperceptible in typical social situations.

Traditional dogma suggests that the submental incision is more easily hidden, but with appropriate placement of the incision in the crux of the neck (cervicomental angle), this incision is well hidden in the shadow of the mandible and heals similarly (Fig. 9). Ultimately, a scar is judged on its visibility. And in the case of a submental versus cervicomental incision, both are imperceptible in social situations, ie, looking at the patient directly rather than from a worm’s eye view (Fig. 10). The added benefit of the cervicomental incision is being its central location, leading to greater access and visibility to the entire neck during surgery (Figs. 9, 10).

Although the SIMI neck lift is a shorter procedure, requiring fewer incisions, the US average cost was approximately the same as a full neck lift, according to the BuildMyBod Health price index.^12^

## CONCLUSIONS

Based on the concept of the Pythagorean theorem, placement of the incision within the cervicomental angle and wide undermining of the neck skin allows the SIMI neck lift to be aggressively used for many types of neck lift patients. Gone are the days when patients with an obtuse cervicomandibular angle must receive unsightly postauricular scars. In the appropriate candidates, the SIMI neck lift provides a minimally invasive option for patients previously only considered a candidate for a full neck lift and may render the traditional mini neck lift obsolete.

## PATIENT CONSENT

*Patients provided written consent for the use of their images*.

## ACKNOWLEDGMENT

The author specially thanks Emily Weiland and Jan Ramiro for the collection of data and article preparation.

## Supplementary Material
