# Supplementary material for: The Single Incision Minimally Invasive (SIMI) Neck Lift
Source: Plast Reconstr Surg Glob Open. 2019 May 16;7(5):e2208. doi: 10.1097/GOX.0000000000002208 (PMC6571294; doi:10.1097/GOX.0000000000002208)
Supplement: Supplementary file 1 [file gox-7-e2208-s001.docx]

January 4, 2019

To Whom It May Concern:

Please see attached original article regarding the single incision minimally invasive (SIMI^™^) neck lift. This article includes a case series, as well as multiple pieces of supplementary digital content submitted separately.

Thanks.

Jonathan Kaplan MD, MPH

Board Certified Plastic Surgeon

Pacific Heights Plastic Surgery

2100 Webster St., Suite 429

San Francisco, CA 94115

415-295-6540

drkaplan@ph-ps.com

**The Single Incision Minimally Invasive (SIMI) Neck Lift**

Author: Jonathan L **Kaplan**, MD, MPH

1. Jonathan L **Kaplan**, MD, MPH. Adjunct Staff – Department of Plastic Surgery in the Dermatology and Plastic Surgery Institute of Cleveland Clinic. Private practice - Pacific Heights Plastic Surgery

**Corresponding Author:**

Jonathan L Kaplan, MD, MPH

2100 Webster St., Suite 429

San Francisco, CA 94115

USA

[drkaplan@ph-ps.com](mailto:drkaplan@ph-ps.com)

Financial Disclosure Statement:

Dr. Kaplan is the founder/CEO of BuildMyBod Health and has ownership equity. No funding was received for this article.

Presented at: AAFPRS Annual Meeting: October 17^th^, 2018 in Dallas, TX

Short running head: Introducing a novel neck lift technique

**Abstract**

Background: With the increased focus on the neck and chin in the era of selfies and social media, neck contouring continues to be an in-demand procedure. Full correction of the neck typically requires both submental and postauricular incisions but the postauricular incisions can be unsightly and painful. This paper introduces a viable alternative for the patient.

Methods: A case series of appropriate candidates undergoing a novel neck lift technique (the single incision minimally invasive (or SIMI) neck lift) is described. Participants include both males and females with an age range of 30-70. The surgical technique incorporates shifting of the typical submental incision used in a full neck lift to a cervicomental incision, and when accompanied by wide undermining, the postauricular incisions are avoided entirely.

Results: Among the 20 patients in this case study, photos show that it’s possible to achieve results commonly associated with a full neck lift, but without the postauricular incisions typically associated with a full neck lift.

Conclusions: The single incision minimally invasive (SIMI) neck lift allows the surgeon to offer an effective procedure for appropriate candidates who want to treat excess fat, a sagging platysma muscle and loose, misplaced skin in the submental region without postauricular incisions typically utilized in a traditional full neck lift.

**Introduction**

Neck contouring continues to be a very sought-after procedure in the era of social media and selfies.^[[1]](#footnote-1)^ Awareness of these procedures via marketing and social media generates interest within the Millennials (born 1981-96, ages 22-37 in 2018) and Generation X (born 1965-80, ages 38-53 in 2018) communities whereas traditionally, patients who consider neck lifts fit within the Baby Boomers (born 1946-64, ages 54-72 in 2018) age bracket.^[[2]](#footnote-2)^

With this changing demographic come different expectations. In addition to seeking out completely non-invasive procedures, they are also considering minimally invasive procedures. Deciding factors include length of work and/or social down time, placement and concealment of incisions, number of incisions and the risk-reward benefit of undergoing the procedure.

To meet the demand of these changing expectations, surgeons must innovate to attract this clientele. In the past, a full neck lift was necessary to achieve long lasting results. However, younger patients are reluctant to have an invasive procedure that requires both submental and post-auricular incisions.

During a cosmetic surgery consultation, the Pythagorean theorem and Euclidean geometry^[[3]](#footnote-3)^ is used as a guide to determine appropriate candidates who will achieve the same results as a traditional neck lift with a novel type of mini-neck lift. This case series discusses the SIMI (single incision minimally invasive) neck lift and how consistent, reproducible results are achievable through one incision instead of three.

**Patients and Methods**

This case series includes 20 consecutive patients that underwent a SIMI (single incision minimally invasive) neck lift. The patients provided informed consent and several patients allowed the primary surgeon and author (JLK) to record the procedure on Snapchat and Instagram as is standard in the author’s practice. Patients also agreed for their photos to be used in this case study and for marketing purposes.

Patients with “misplaced” midline neck skin, without a great deal of excess skin, were considered candidates for this modified neck lift. The 20 patients included in this study underwent surgery between May of 2015 and August of 2018 (approximately 3.5 years). They were fairly evenly divided between males and females (11 women, 9 men) with an average age of 55 years old (range 30-70). (Table 1)

**Operative Technique (SDC1)**

In the preoperative holding area, the patient is marked. A 3-4cm line is drawn on the radial side of the left index finger and then superimposed on the junction of the horizontal and vertical surface of the neck (the cervicomental angle) in a transverse direction. Using this technique allows the surgeon to feel for that junction, thus ensuring the incision is placed in the most hidden, concave surface of the cervicomental angle of the neck, within the neck’s natural shadow. (See video, Supplemental Digital Content 1 which shows appropriate preoperative marking of the ideal SIMI neck lift candidate, intraoperative technique and reproducible postoperative results. THIS VIDEO IS AVAILABLE IN THE “RELATED VIDEOS” SECTION OF THE FULL-TEXT ARTICLE ON PRSJOURNAL.COM OR AT http://links.lww.com/PRSGO/B71)

The lateral extent of the neck dissection is marked along the edge of the sternocleidomastoid, with the superior most dissection marked along the jawline and the inferior most dissection marked along the base of the neck. The 3-4cm incision allows full access for complete dissection of these areas.

In the operating room, the patient is given a standard dose of 1 gram of cefazolin and SCD’s are placed. This is a procedure that can be done under both conscious sedation or general anesthesia.

A stab incision is made in the center of the transverse preop marking in the cervicomental angle of the neck with a #15 blade. 250cc of tumescent solution is infiltrated under low flow. Tumescent solution consists of saline and 1 amp of epinephrine (no lidocaine) if under general or 50cc of 1% lidocaine and 1 amp of epinephrine if performed under conscious sedation. After infiltration, 7-10 minutes is allowed to elapse for maximum vasoconstriction.

Liposuction with a 3mm cannula is performed along the entirely of the neck, extending to the sternocleidomastoid and especially along the jawline to reduce any jowling that is present. After liposuction, the remainder of the transverse neck incision is completed.

Under direct visualization with a lighted retractor, the neck skin is elevated bilaterally to the sternocleidomastoid, anteriorly towards the chin and inferiorly towards the base of the neck. Because there are no postauricular incisions in the SIMI neck lift, the surgeon must widely undermine and reposition the misplaced skin since there will not be an opportunity to remove excess skin via postauricular incisions, since these are avoided in this technique.

After elevation and hemostasis, the medial edges of the platysma are delineated in preparation for the corset platysmaplasty as described by Feldman.^[[4]](#footnote-4)^ Any subplatysmal fat is excised down to the level of the digastric muscles. A 4-0 PDS is run from the superior aspect of the platysma, to the base of the neck and back up in a running horizontal mattress fashion to minimize the appearance or palpation of a midline ridge.

Any excess fat present just posterior to the chin or attached to the undersurface of the skin flap is directly excised. One 10 French round drain is brought through a separate stab incision behind either earlobe and placed across the entire neck. Only one drain is used and is sewn into place with leftover 4-0 PDS used in the corset platysmaplasty.

The cervicomental incision is closed with 6-0 fast absorbing gut along the skin edges in a baseball stitch fashion (simple continuous or running suture technique). No dermal stitch is required. The patient is placed into a postop head and neck garment and transferred to the recovery room. Patients are sent home with an extra garment and an already-filled prescription of pain medication, anti-emetics and silicone scar cream. The silicone scar cream can be applied 2 weeks after procedure and is recommended to further minimize the appearance of the scar by increasing collagen production.^[[5]](#footnote-5)^

The patient is seen the next morning (postoperative day one) to remove the drain. Showers commence the next morning after drain removal. The garment is worn all of the time during the first week except during showers, and then only at night for several more weeks.

Because the only incision is closed with absorbable stitches, the patient does not have to return for suture removal, other than the drain stitch on postoperative day one. The patient returns for a checkup and postop photos at 2 weeks and 3-4 months.

**Results**

Twenty patients underwent a SIMI neck lift. These were all primary cases. Patients that were considered candidates for the SIMI neck lift were those with excess fat and skin to the neck and reflect the SIMI neck lift pathway in the decision tree found in Figure 8.

The only observed complication was an immediate postop hematoma that was noted in the recovery room. The patient was brought back to the operating room and the operative site was explored through the existing cervicomental incision. No vessel was identified other than generalized ooze. Hemostasis was achieved without the need for additional incisions, and the patient had an uncomplicated recovery from that point onward.

One patient had residual submental fat remaining after his SIMI neck lift. Dissolution of the fat with deoxycholic acid was offered but the patient was happy with his result and declined.

Each figure shows results at 3-4 months postop. The impetus for doing a SIMI neck lift, or why a SIMI neck lift was more appropriate than another technique is clarified with each before and after result.

**Case #1**

The patient in case #1 was a perfect candidate for a SIMI neck lift because he had minimal excess skin and fat. However, a lax platysma accentuated the excess skin and fat, giving him a “double chin” appearance.

Due to this lax platysma, liposuction alone would not achieve his desired result. A traditional mini neck lift through a submental incision would not allow for the wide undermining necessary to redrape the skin and once the underlying platysma muscle is tightened, the skin would continue to hang or buckle along the edge of the non-undermined areas. A full neck lift requiring postauricular incisions would achieve the same result as achieved by the SIMI neck lift. But with no excess skin to treat or remove, the additional postauricular incisions would have been unnecessary and too visible with the patient’s short hair.

As seen in Figure 1, the patient’s results improved during the first 3 months post-op as the redraped skin continued to tighten. His chin appears more prominent and he no longer has the “double chin” appearance.

**Case #2**

The SIMI neck lift was appropriate for patient #2 because she had “misplaced” midline skin.

She did not have excess fat so liposuction alone would not have adequately treated her. Some skin redraping would occur with liposuction alone but the surgeon has greater control in skin redraping by manually undermining skin rather than depending on the redraping from liposuction alone. And because this patient has misplaced midline skin, not excess skin, skin excision was not necessary and therefore, postauricular incisions were not necessary.

As seen in Figure 2, the patient’s jawline is more well-defined after being treated with the SIMI neck lift. Once the skin was undermined and redraped, the skin is no longer loose and the neck appears thinner. The transverse lines present pre and postop are natural etched-in lines that are not treated with any neck lift technique.

**Case #3**

Patient #3 was a perfect candidate for a SIMI neck lift for reasons similar to the Patient #1. A very lax platysma and excess midline skin obscured the patient’s thyroid cartilage, a hallmark of masculinity. This contour was restored with his procedure.

As a male patient with short hair, postauricular incisions are difficult to hide. Performing a SIMI neck lift to redrape the skin through a cervicomental incision was most appropriate in avoiding postauricular incisions. As stated previously, the submental incision used in the traditional mini neck lift would not allow for wide undermining necessary for adequate skin redraping.

Similar to Patient #1, as seen in Figure 3, once the patient’s platysma was tightened and the hanging skin in the neck was undermined and redraped, the contours of his neck became more prominent. Before the surgery, the patient’s thyroid cartilage was not visible and the redraping of the skin exposed it.

This patient had residual submental fat after his procedure but he declined to do anything further since he was pleased with his result.

**Case #4**

Patient #4 had a significant amount of midline excess skin. This patient could easily be a candidate for a full neck lift utilizing a submental incision and postauricular incisions. While she could more easily cover incisions behind the ears with longer hair, she did not want the pain associated with postauricular incisions. Therefore, she opted for a SIMI neck lift.

With so much excess skin, she was at risk of wrinkling or folding of the redraped skin if there was not adequate recoil and tightening. At the 2-week mark, she did indeed have a fold of skin resulting in a crease but this resolved over time and is no longer present in her 3 month post-operative photo as seen Figure 4. There are subtle striations seen on the skin surface but there are no folds, wrinkles or creases remaining.

**Case #5**

Patient #5 had hanging skin that covered his laryngeal prominence. Because this skin was not in excess, he was a candidate for a SIMI neck lift. Redraping of the skin gave him more prominent chin profile. Additionally, his laryngeal prominence is more noticeable. (Figure5)

**Case #6**

Patient #6 was a candidate for a SIMI neck lift because he had loose skin underneath his chin and on the lateral sides of his neck starting below his ears. Since this loose skin had minimal fat, he would not be a candidate for liposuction alone. The wide undermining of the skin during the SIMI neck lift procedure allowed all of the misplaced, bunched-up areas of skin to be redraped and smoothed out, giving his neck a more youthful appearance. (Figure 6)

**Discussion**

The introduction of a new technique such as the SIMI neck lift^[[6]](#footnote-6)^ requires a review of the “benchmark” procedures that it builds upon (Table 2). This also requires an agreement on the terminology used when referring to those benchmark procedures. For example, we cannot reference a full neck lift and a mini neck lift without clarifying what those procedures entail, since those terms mean different things to different surgeons. And while we won’t necessarily agree on the terminology in every context, we can agree on the terminology, at least temporarily, within the confines of this case series.

Therefore, for the sake of this discussion, a full neck lift refers to a procedure wherein incisions are made to the submental and postauricular areas followed by liposuction, submental fat resection, wide undermining of skin, corset platysmaplasty and skin excision from the postauricular area.

A mini neck lift is more conservative by nature. This procedure as described by Knize requires a submental incision, just behind the chin, to remove submental fat and possibly a corset platysmaplasty.^[[7]](#footnote-7)^

Based on this terminology, the SIMI neck lift technique is different in regard to:

1. Incision placement: In the SIMI neck lift, the incision is more posterior, moving the traditional incision from a submental to a cervicomental location to allow greater access to the neck
2. Much more extensive undermining of the skin. The goal of the SIMI neck lift, compared to the mini neck lift, is the redraping of the “misplaced” skin. Moving the submental incision to a cervicomental position in a SIMI neck lift allows for the wide undermining achieved during a full neck lift without requiring any postauricular incisions.

Using the terminology, “misplaced,” is meant to describe a normal amount of skin that is simply misplaced in the midline of the neck. By redraping the skin, it is repositioned to a more aesthetically pleasing position. Since there is not “excess, hanging” skin, postauricular incisions are not needed to excise this skin. (Table2)

**The Pythagorean Theorem as part of the SIMI neck lift**

To rely solely on skin redraping requires an understanding of the Pythagorean theorem when choosing appropriate candidates for the SIMI neck lift. As seen in Figure 7, the right angle triangle is labeled x and y to represent the two shorter sides of the triangle (the catheti), connected by the hypotenuse, h, the longer line opposite the right angle. x represents line segment $\bar{AB}$, y represents line segment $\bar{AC}$ and h represents line segment $\bar{BC}$. $\angle BAC$ is the right angle and is equal to 90°. Based on the Phythagorean theorem, the square of the two catheti is equal to the square of the hypotenuse, $x^{2}+y^{2}=h^{2}$. Therefore, the sum of the two catheti is always greater than the length of the hypotenuse, $x+y>h$.

Based on this geometry, any “misplaced” or “hanging” skin that is equal to or less than the hypotenuse, then this skin can be accommodated within the natural contours of the neck, in line with the two catheti. When the right angle triangle is superimposed onto the neck as seen in Figure 8 below, x and y represent the mandibular and cervical portions respectively of the neck and the hypotenuse represents the lax, obtuse cervicomandibular angle.

Furthermore, the Vertex a is where the incision is placed at the crux of the neck or the junction of the horizontal and vertical surface of the neck (the cervicomental angle). Additionally, since this is the location of the transverse incision, this gives the surgeon the most access for the wide undermining described in the operative technique. Vertex b is located at the mandibular prominence to form the imaginary line segment $\bar{AB}$. Lastly Vertex c is placed at the suprasternal notch to form the imaginary line segment $\bar{AC}$. From these two points, the imaginary line segment $\bar{BC}$ can be drawn to form our hypotenuse. (Figure 7)

As previously discussed, based on the Pythagorean theorem, the sum of the lengths of the two catheti is always greater than the length of the hypotenuse,$x+y>h$. Therefore, in appropriate candidates when the cervicomandicular angle aligns with the hypotenuse, the lax skin (the hypotenuse in this analogy) can be redraped and accommodated within the limbs (the catheti) overlying the mandibular and cervical portions of the neck. When represented by an equation, a patient is an excellent candidate for a SIMI neck lift when:$x+y>h$. As seen in Figure 8 above, if the excess (or misplaced) skin does not pass the hypotenuse line of the superimposed right triangle, the patient’s natural neck contours can smoothly accommodate the skin once undermined and redraped. When candidates met these criteria, they were recommended for the SIMI neck lift.

However, if the patient has a great deal of excess skin wherein the cervicomandibular angle (hypotenuse) is greater than the mandibular and cervical limbs of the right-angle triangle; ie, the $x+y<h$, this type of patient is **not** a candidate for the SIMI neck lift because the excess skin cannot be accommodated along the jawline and neck. A patient with excess skin that hangs past the line of the hypotenuse of this right triangle would require skin excision and would be a more appropriate candidate for a full neck lift.

As shown in Figure 8, patients can be separated into varying treatment pathways based on skin excess, fat excess or platysmal banding. Patients with supraplatysmal fat only (no excess skin) are candidates for liposuction alone.^[[8]](#footnote-8)^ Patients with subplatysmal fat, platysmal banding or redundant skin can be further divided based on excess skin (direct excision with Z-plasty as described by Zins or a full neck lift)^[[9]](#footnote-9),^^[[10]](#footnote-10)^ or misplaced skin (SIMI neck lift).

**Benefits of the SIMI neck lift**

When compared to the full neck lift, the SIMI neck lift avoids painful or unsightly postauricular scars. While less painful incisions benefit both males and females, the lack of scars behind the ears are particularly beneficial for men since they typically have short-cropped hair, making it more difficult to hide incisions in this area. While pain from postauricular incisions can vary between surgical technique and a patient’s pain threshold, clearly the *absence* of a postauricular incision will lead to less pain than the *presence* of a postauricular incision.

With only a single cervicomental incision, this is clearly a less invasive procedure compared to its “full neck lift” counterpart. The more posteriorly placed cervicomental incision provides easier access to the entire neck, making wide skin undermining easier. Performing the entire operation through the cervicomental incision avoids not only post auricular incisions but also the need for postauricular skin flap elevation. Wide undermining and single incision placement are the key distinguishing factors of the SIMI neck lift when compared to the previously described, “limited incision submental lipectomy and platysmaplasty” by Knize.^[[11]](#footnote-11)^7

In regard to the Knize cohort, it’s important to note that our cohort of 20 patients over 3.5 years (5.7 patients per anum) is comparable to his cohort of 56 patients over 15 years (3.7 patients per anum).

Additional benefits of the SIMI neck lift include fewer incisions which can lead to shorter operative times due to less time required to close postauricular incisions. A reasonable assumption is that fewer incisions will lead to less pain and therefore a shorter recovery when compared to the full neck lift. Certainly, a prospective cohort study can further evaluate these potential advantages.

Traditional dogma suggests the submental incision is more easily hidden, but with appropriate placement of the incision in the crux of the neck (cervicomental angle), this incision is well hidden in the shadow of the mandible and heals similarly (Figure 9). Ultimately, a scar is judged on its visibility. And in the case of a submental vs cervicomental incision, both are imperceptible in social situations, ie looking at the patient directly rather than from a worm’s eye view (Figure 10). The added benefit of the cervicomental incision being it’s central location, leading to greater access and visibility to the entire neck during surgery. (Figure 9-10)

Although the SIMI neck lift is a shorter procedure, requiring fewer incisions, the US average cost was approximately the same as a full neck lift, according to the BuildMyBod Health price index.^[[12]](#footnote-12)^

**Conclusions**

Based on the concept of the Pythagorean theorem, placement of the incision within the cervicomental angle and wide undermining of the neck skin allows the SIMI neck lift to be aggressively used for many types of neck lift patients. Gone are the days when patients with an obtuse cervicomandibular angle must receive unsightly postauricular scars. In the appropriate candidates, the SIMI neck lift provides a minimally invasive option for patients previously only considered a candidate for a full neck lift and may render the traditional mini neck lift obsolete.

**Legend**

**Figure 1: Patient #1 before and after results at 3 months**

**Figure 2: Patient #2 before and after results at 3 months**

**Figure 3: Patient #3 before and after results at 3 months**

**Figure 4: Patient #3 before and after results at 3 months**

**Figure 5: Patient #5 before and after results at 4 months**

**Figure 6: Patient #6 before and after results at 6 weeks**

**Figure 7: Right angle triangle superimposed on neck**

**Figure 8: SIMI neck lift decision tree**

This decision tree delineates which patients are candidates for a full neck lift, liposuction only, direct excision with Z-plasty or the SIMI neck lift. Image showing the test for supra vs subplatysmal fat is used courtesy The Art of Aesthetic Surgery, 1^st^ Edition, Foad Nahai, ed. Thieme.^[[13]](#footnote-13)^

**Figure 9: SIMI neck lift incision**

The SIMI neck lift incision is hidden in the cervicomental angle of the neck. Here is an example of its appearance 15 months after a SIMI neck lift. The scar is comparable to the patient’s much older submental incision from her facelift 20 years prior, by another surgeon. But most importantly, this worm’s eye view is not the view of the neck seen in everyday social situations.

**Figure 10: Frontal view of SIMI neck lift incision at 3-4 months postoperatively**

Frontal view of all 6 patients highlighted in this case series, showing that the cervicomental incision is imperceptible in typical social situations.

**SDC 1** – See video, Supplemental Digital Content 1 which shows appropriate preoperative marking of the ideal SIMI neck lift candidate, intraoperative technique and reproducible postoperative results. THIS VIDEO IS AVAILABLE IN THE “RELATED VIDEOS” SECTION OF THE FULL-TEXT ARTICLE ON PRSJOURNAL.COM OR AT INSERT LINK HERE

**Patient consent**

*Patients provided written consent for the use of their images.*

**Acknowledgement**

*Special thanks to Emily Weiland and Jan Ramiro for the collection of data and manuscript preparation.*

**References**

Ledwith, M. 2016 Oct 23. Selfie fans turn to surgery to get the perfect picture. Retrieved from <http://www.dailymail.co.uk/news/article-3865358/Selfie-fans-turn-surgery-perfect-picture-Growing-numbers-opting-operations-rid-double-chin.html>

^2^ Dimock, M. 2018 Mar 1. Defining generations: Where Millennials end and post-Millennials begin. *Pew Research Center*. Washington, D.C. Retrieved from <http://www.pewresearch.org/fact-tank/2018/03/01/defining-generations-where-millennials-end-and-post-millennials-begin/>

^3^ Morris, SJ. The Pythagorean Theorem. *The University of Georgia Department of Education*. Retrieved from <http://jwilson.coe.uga.edu/emt669/student.folders/morris.stephanie/emt.669/essay.1/pythagorean.html>

^4^ Feldman, JJ. Corset platysmaplasty. *Plast. Reconstr. Surg*. 85: 333, 1990.

^5^ Kim, S., Choi, J., Lee, J., Kim, Y., Jun, Y. Prevention of Postsurgical Scars: Comparison of Efficacy and Convenience between Silicone Gel Sheet and Topical Silicone Gel. *J Korean Med Sci*. 29: S249-S253.

^6^ Kaplan JL. The SIMI^TM^ Neck Lift. Retrieved from <https://www.pacificheightsplasticsurgery.com/procedures/neck-lift/>

^7^ Knize, DM. Limited incision submental lipectomy and platysmaplasty. *Plast. Reconstr. Surg.* 101: 473, 1998.

^8^ Gryskiewicz, JM. Submental suction-assisted lipectomy without platysmaplasty. *Plast. Reconstr. Surg*. 112: 1393, 2003.

^9^ Zins, JE; Fardo, D. The “Anterior Only” Approach to Neck Rejuvenation: An Alternative to Face Lift Surgery. *Plast. Reconstr. Surg. 115: 1761-1768, 2005.*

^10^ Zins, JE, Menon N. Anterior approach to neck rejuvenation. *Aesthet Surg J.* 2010; 30: 477-484.

^11^ The Art of Aesthetic Surgery, 1^st^ Edition. Foad Nahai ed. Theime publishing.

^12^ BuildMyBod Price Index, accessed at [www.buildmybod.com/providers](http://www.buildmybod.com/providers) on August 13, 2018.

1. Ledwith, M. 2016 Oct 23. Selfie fans turn to surgery to get the perfect picture. Retrieved from <http://www.dailymail.co.uk/news/article-3865358/Selfie-fans-turn-surgery-perfect-picture-Growing-numbers-opting-operations-rid-double-chin.html> [↑](#footnote-ref-1)
2. Dimock, M. 2018 Mar 1. Defining generations: Where Millennials end and post-Millennials begin. *Pew Research Center*. Washington, D.C. Retrieved from <http://www.pewresearch.org/fact-tank/2018/03/01/defining-generations-where-millennials-end-and-post-millennials-begin/> [↑](#footnote-ref-2)
3. Morris, SJ. The Pythagorean Theorem. *The University of Georgia Department of Education*. Retrieved from <http://jwilson.coe.uga.edu/emt669/student.folders/morris.stephanie/emt.669/essay.1/pythagorean.html> [↑](#footnote-ref-3)
4. Feldman, JJ. Corset platysmaplasty. *Plast. Reconstr. Surg*. 85: 333, 1990. [↑](#footnote-ref-4)
5. Kim, S., Choi, J., Lee, J., Kim, Y., Jun, Y. Prevention of Postsurgical Scars: Comparison of Efficacy and Convenience between Silicone Gel Sheet and Topical Silicone Gel. *J Korean Med Sci*. 29: S249-S253. [↑](#footnote-ref-5)
6. Kaplan JL. The SIMI^TM^ Neck Lift. Retrieved from <https://www.pacificheightsplasticsurgery.com/procedures/neck-lift/> [↑](#footnote-ref-6)
7. Knize, DM. Limited incision submental lipectomy and platysmaplasty. *Plast. Reconstr. Surg.* 101: 473, 1998. [↑](#footnote-ref-7)
8. Gryskiewicz, JM. Submental suction-assisted lipectomy without platysmaplasty. *Plast. Reconstr. Surg*. 112: 1393, 2003. [↑](#footnote-ref-8)
9. Zins, JE; Fardo, D. The “Anterior Only” Approach to Neck Rejuvenation: An Alternative to Face Lift Surgery. *Plast. Reconstr. Surg. 115: 1761-1768, 2005.* [↑](#footnote-ref-9)
10. Zins, JE, Menon N. Anterior approach to neck rejuvenation. *Aesthet Surg J.* 2010; 30: 477-484. [↑](#footnote-ref-10)
11. 7 Knize, DM. Limited incision submental lipectomy and platysmaplasty. *Plast. Reconstr. Surg.* 101: 473, 1998. [↑](#footnote-ref-11)
12. BuildMyBod Price Index, accessed at [www.buildmybod.com/providers](http://www.buildmybod.com/providers) on August 13, 2018. [↑](#footnote-ref-12)
13. The Art of Aesthetic Surgery, 1^st^ Edition. Foad Nahai ed. Theime publishing. [↑](#footnote-ref-13)
